# Applicability of Pulse Pressure Variation during Unstable Hemodynamic Events in the Intensive Care Unit: A Five-Day Prospective Multicenter Study

**DOI:** 10.1155/2016/7162190

**Published:** 2016-03-31

**Authors:** Bertrand Delannoy, Florent Wallet, Delphine Maucort-Boulch, Mathieu Page, Mahmoud Kaaki, Mathieu Schoeffler, Brenton Alexander, Olivier Desebbe

**Affiliations:** ^1^Service de Réanimation Médicale, Hôpital de la Croix-Rousse, Hospices Civils de Lyon, Lyon, France; ^2^Service de Réanimation, Centre Hospitalier Lyon-Sud, Hospices Civils de Lyon, Pierre-Bénite, France; ^3^Hospices Civils de Lyon, Service de Biostatistique, Lyon, France; ^4^CNRS, UMR5558, Laboratoire de Biométrie et Biologie Evolutive, Equipe Biostatistique-Santé, Université Lyon 1, 69100 Villeurbanne, France; ^5^Department of Anesthesiology and Critical Care Medicine, Pavilion P, Edouard Herriot Hospital, Hospices Civils de Lyon, Claude Bernard University Lyon 1, 69003 Lyon, France; ^6^Intensive Care Unit, Centre Hospitalier de Roanne, 42300 Roanne, France; ^7^Unité de Réanimation Chirurgicale, Hospices Civils de Lyon, Hôpital de la Croix-Rousse, Université de Lyon, 69004 Lyon, France; ^8^Department of Anesthesiology & Perioperative Care, School of Medicine, University of California, Irvine, Orange, CA 92868, USA; ^9^Department of Anesthesiology and Intensive Care, Clinique de la Sauvegarde, 69009 Lyon, France; ^10^Université Lyon 1, EA4169, SFR Lyon-Est Santé, INSERM US 7, CNRS UMS 3453, Lyon, France

## Abstract

Pulse pressure variation can predict fluid responsiveness in strict applicability conditions. The purpose of this study was to describe the clinical applicability of pulse pressure variation during episodes of patient hemodynamic instability in the intensive care unit. We conducted a five-day, seven-center prospective study that included patients presenting with an unstable hemodynamic event. The six predefined inclusion criteria for pulse pressure variation applicability were as follows: mechanical ventilation, tidal volume >7 mL/kg, sinus rhythm, no spontaneous breath, heart rate/respiratory rate ratio >3.6, absence of right ventricular dysfunction, or severe valvulopathy. Seventy-three patients presented at least one unstable hemodynamic event, with a total of 163 unstable hemodynamic events. The six predefined criteria for the applicability of pulse pressure variation were completely present in only 7% of these. This data indicates that PPV should only be used alongside a strong understanding of the relevant physiology and applicability criteria. Although these exclusion criteria appear to be profound, they likely represent an absolute contraindication of use for only a minority of critical care patients.

## 1. Background

Hemodynamic instability is a common indication for ICU admission and is very likely to occur during a typical ICU stay. It is also well accepted that fluid loading to increase cardiac output (CO) is a major component of appropriate resuscitation [1]. Unfortunately, this intervention has been shown to be harmful when hemodynamically unnecessary [2]. Therefore, it would be extremely valuable to be able to predict an increase in CO before administering fluid. This has been attempted for years using static indices such as central venous pressure (CVP), although it is now known that these are poorly predictive of a patient's response to volume expansion [3]. Variables calculated using cardiopulmonary interactions with mechanical ventilation, termed “dynamic indices,” have been increasingly developed in the past 20 years [4]. One of the most popular indices is pulse pressure variation (PPV). PPV has been shown to predict fluid responsiveness in the ICU with good sensitivity and specificity under appropriate clinical conditions [5]. Additionally, PPV can be displayed automatically and continuously.

Unfortunately, a wide variety of physiologic inputs to the cardiopulmonary system will influence PPV, indicating that this variable is limited to very strict clinical conditions (specific contraindication listed in Conditions for PPV Applicability in Material and Method). Studies have found that a significant range (2 to 46%) of patients in the ICU actually meet the criteria for the appropriate use of PPV during a 24-hour period [6, 7]. However, when addressing the applicability of such a dynamic index, one must remember that a patient's clinical condition is a continuously changing process and cannot be summarized using simply demographic and static data. As such, no prospective study has evaluated the applicability of PPV in the ICU with regard to specific real-time periods of hemodynamic instability, during which such knowledge would be most valuable in optimizing patient care. Therefore, the goal of this study was to prospectively assess the percentage of critical unstable hemodynamic events (UHE) in which the use of PPV is valid.

## 2. Material and Method

### 2.1. Ethical Considerations

The institutional ethics committee (Comité d'Ethique des Hospices Civils de Lyon, Lyon, France) approved this trial on December 1st, 2010. Our study was considered to be strictly observational and thus the need for written informed consent was waived. Patients or their next of kin were verbally informed about their participation to the study and given the option to refuse.

### 2.2. Inclusion Centers

We performed a multicenter prospective study that included seven ICUs within 5 hospitals. There were a total of 92 beds included from four surgical units, two medical units, and one mixed unit.

### 2.3. Inclusion Criteria

Any patient over 18 years of age presenting with an acute unstable hemodynamic event (UHE) was included. An UHE was defined by the presence of at least one of the following criteria: (1) arterial systolic blood pressure under 90 mmHg (or greater than 50 mmHg decrease in a previously hypertensive patient) or the need of vasopressor or inotropic drugs to maintain a systolic blood pressure over 90 mmHg; (2) urine output under 0.5 mL/kg/h for more than 2 hours; (3) tachycardia (heart rate over 100/min); (4) presence of skin mottling. Although not all independently validated, we used these four inclusion criteria as they were the variables described in the original PPV article by Michard et al. [8].

### 2.4. Inclusion Period

Patients were included over a predetermined period of five consecutive days during November 2011. A maximum of one UHE per day per patient was recorded over the five-day inclusion period. UHE were further characterized as septic, cardiogenic, or “other” (hypovolemic, obstructive, or other distributive factors) [2].

### 2.5. Conditions for PPV Applicability

Seven clinical requirements for PPV applicability were screened in the participating patients at the time of each UHE: (1) mechanical ventilation; (2) patient under sedation without any spontaneous respiratory movement [8]; (3) mechanical ventilation with tidal volume (TV) >7 mL/kg of ideal body weight (IBW) [9]; (4) ratio of heart rate (HR) to respiratory rate (RR) greater than 3.6 [10]; (5) sinus rhythm; (6) no significant heart valve disease; (7) no echography evidence of right ventricular failure (defined by paradoxical interventricular septal motion) [11].

### 2.6. Statistical Analysis

The main outcome was appropriate PPV applicability defined as patients meeting all of the above requirements. Data were described with median and first and third quartiles for continuous variables and raw values and percentages for categorical variables.

## 3. Results

### 3.1. Unstable Hemodynamic Events

Over the five-day inclusion period, 164 patients were screened which corresponded to a total of 487 hospital days. Seventy-five (46%) patients presented with at least one UHE during the five-day inclusion period. Two patients were excluded: one due to refusal and one due to missing data. Among the 73 patients presenting an UHE, the total number of UHE was 163. The median number of recorded events per patient was 2 [1–3]. Demographic data are presented in Table 1.

### 3.2. Applicability of Conditions for PPV

**Figure 1:**
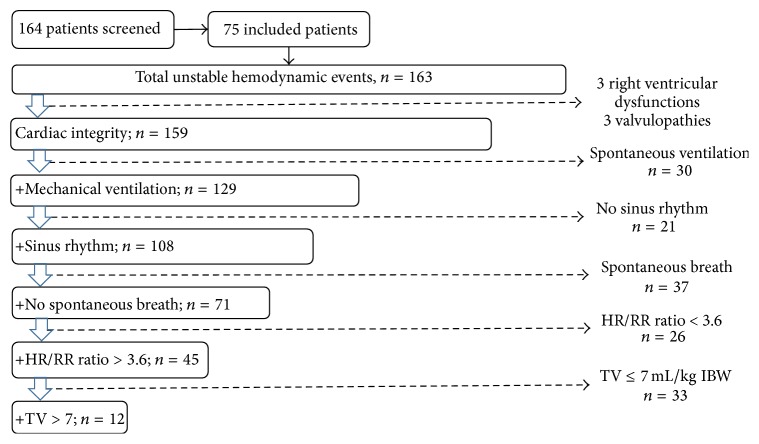
Flow chart showing pulse pressure variation applicability according to the accumulation of applicability criteria. This flow chart shows that one chosen criterion includes 159 UHE, two criteria include 129 UHE, three criteria include 108 UHE, four criteria include 71, five criteria include 45, and all criteria include 12 UHE. This figure systematically evaluates a large group of patients by exclusion criteria, and once a patient was excluded no further criteria were considered for that patient. We used this format to describe how a clinician might systematically approach the use of PPV in a patient. HR: heart rate; RR: respiratory rate; TV: tidal volume; UHE: unstable hemodynamic events.

**Figure 2:**
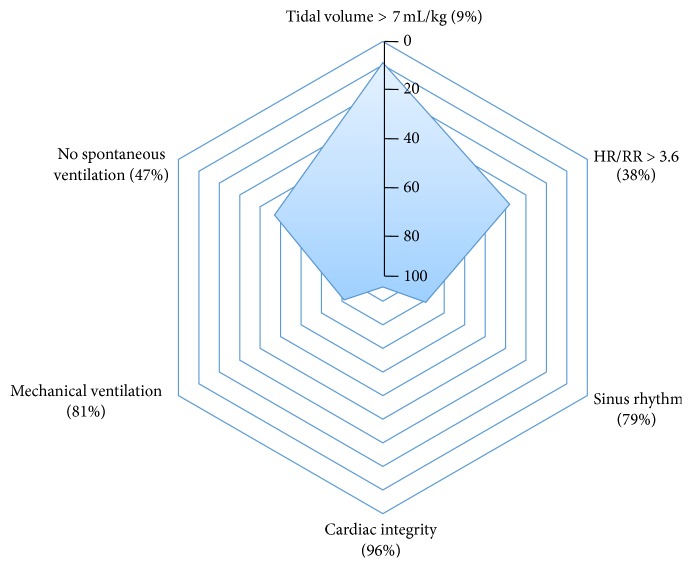
Radar chart showing the PPV applicability criteria according to the total number of UHE. The represented inverse scale allows one to observe the minimal presence of a tidal volume >7 mL/kg IBW, especially in relation to other PPV contraindications. For instance, this chart shows that, in 81% of UHE, patients were mechanically ventilated.

Among the 163 UHE, 12 events (7%) fulfilled the seven conditions for the appropriate application of PPV, with 10 of these events corresponding to patients admitted to the ICU for postoperative care.  Figure 1 shows the flow chart for PPV applicability according to the total number of UHE. Total frequency of applicability of PPV for each of the exclusion criteria is depicted in Figure 2. Low TV was the most frequent limitation of PPV applicability with a TV >7 mL/kg present in only 9% of UHE.

## 4. Discussion

This study is the first to prospectively evaluate the applicability of PPV during real-time unstable hemodynamic events in the ICU. The results demonstrate that only 7% of these UHE strictly meet the PPV applicability criteria, the majority being patients coming from the operating room. As previously stated, PPV has been shown to predict fluid responsiveness (and can therefore be used to optimize fluid management) only if patients meet appropriate criteria [5]. In earlier ICU applicability studies, situational applicability of PPV was assessed using data from a completed 24-hour period [6, 7]. In contrast, the present study was done over a five-day period and evaluated PPV applicability during real-time events and not according to baseline characteristics of all ICU patients. As a result of this design, we believe that our analysis more accurately addresses the critical scenarios in which optimal fluid titration would be most beneficial. For example, compared to a recent one-day study [7], our population was more critically ill, with a median [interquartile range] SOFA score of 7 [6–11] versus 4 [3-4]. This difference likely explains our populations increased requirements for mechanical ventilation (81% versus 51%).

Conversely, with respect to overall PPV applicability, our conclusions are quite similar to other recent publications, due mostly to the exclusion of patients with low TV (91% of the UHE, representing 92% of the patients). The low TV being used are based on promising results in ARDS patients [12] despite the fact that clinical benefit of low TV in non-ARDS ICU patients remains unclear [13]. For this population one must consider a transient increase in TV to allow for sufficient cardiopulmonary interactions to measure PPV, as this would have increased the PPV applicability rate from 7% to 33% of UHE. Additionally, it must be stressed that a low TV does not mean that PPV should not be used, but it rather should be used with caution, as the risk of false negative values increases [14]. As described by Mesquida et al., in low TV conditions, high PPV values remain reliable to preload responsiveness [15]. Thus, high PPV patients should be considered for fluid challenge regardless of their TV.

Interestingly, our study demonstrated that postoperative patients are by far the most likely to meet the applicability criteria. This is a clinical situation where multimodal hemodynamic monitoring, including dynamic indices, has a strong potential to decrease postoperative complications [16]. As TV is usually greater in the OR, it is possible that ventilation settings were kept constant during transitions to postoperative care. However, this proportion will likely decrease even further with the recently proven benefits of a respiratory protective strategy in the OR [17].

### 4.1. Study Limitations

The small sample size of this study combined with a disproportionately high rate of postoperative patients (32%) may not accurately represent the wide variety of patients in all available ICUs. Additionally, we chose to use clinical UHE criteria, although biological markers (lactate, mixed venous oxygen saturation, etc.) could have refined the number of included patients. As PPV can increase before any variation in conventional hemodynamic variables (such as those used for our current definition of an UHE), PPV values could have been retrospectively examined at the time before the UHE to determine if it was able to predict these episodes. Unfortunately, PPV was not continuously monitored, and we therefore cannot answer this question in the current study.

As one UHE can last from a few minutes to several hours, we deliberately included only one UHE per day, which may be seen as a weakness. This decision was felt justified as defining the transition zone between two UHE in such a short period of time would have significantly confounded the results. Additionally, contraindications for the use of PPV could have included more than the seven described here, as low pulmonary compliance [18], high left filling cardiac pressure [19], and low heart rate [20] have all shown moderate clinical evidence. Additionally, right ventricular dysfunction and intra-abdominal hypertension can allow for false positive values [11]. As can be seen by the wide variety of available inclusion and exclusion criteria, proposing fixed clinical criteria for standardized hemodynamic optimization using PPV would decrease potential clinical confusion in the future. Beyond these additional hemodynamic criteria, other limitations intrinsic to the measurement and interpretation of PPV are emerging. Firstly, the question of how PPV is actually measured has been highlighted, with some data indicating that a long averaging time can decrease the predictive value [21]. Secondly, the grey zone of PPV in the ICU can vary from 4 to 17%, which affects 62% of patients [22]. This data, combined with our findings, may lead physicians without extensive hemodynamic training to consider other functional tests to assess fluid responsiveness, such as a fluid challenge or passive leg raising [23]. However, both are time consuming and require a relevant cardiac output monitor or an echographic trained practitioner. A final limitation of our work concerns cardiogenic shock patients wherein PPV has not been adequately described. Mesquida et al. did demonstrate that cardiac contractility alters PPV reliability, and without further data the validity of PPV in cardiogenic shock condition remains controversial [15].

## 5. Conclusion

Unstable hemodynamic states affect almost half of ICU patients. In this five-day ICU multicenter prospective study, PPV was determined to be strictly applicable in only 7% of such UHE, with most of them being in postoperative patients. A protective ventilation strategy with low tidal volume was the main limiting factor. This data indicates that PPV should only be used alongside a strong understanding of the relevant physiology and applicability criteria. Although these exclusion criteria appear to be profound, they likely represent an absolute contraindication of use for only a minority of critical care patients.

## Figures and Tables

**Table 1:** Patient background characteristics *n* = 73.

Age (years)	63 (54–74)
Gender (M/F) (%)	68/32
SAPS II	49 (37–62)
Reasons for admission (%)	
Postoperative care	32
Cardiac surgery	23
General surgery	9
Nonpostoperative care	68
Respiratory failure	25
Sepsis	22
Circulatory failure	8
Neurological disorder	8
Other	5

Values are presented as “median values (first quartile–third quartile)” or percentage of total.

F: female; M: male; SAPS II: simplified acute physiology score II.
